# Patients with immune mediated inflammatory diseases are insufficiently protected against vaccine-preventable infections

**DOI:** 10.1007/s15010-024-02373-z

**Published:** 2024-08-22

**Authors:** Natasja van de Pol, C. Janneke van der Woude, Marijn Vis, Martijn B.A. van Doorn, Saskia L. Schrauwen, Fatos Cetinözman-Teunissen, Rachel L. West, Annemarie C. de Vries

**Affiliations:** 1 Department of Gastroenterology and Hepatology, Erasmus University Medical Center, Rotterdam, the Netherlands; 2 Department of Rheumatology, Erasmus University Medical Center, Rotterdam, the Netherlands; 3 Department of Dermatology, Erasmus University Medical Center, Rotterdam, the Netherlands; 4 Department of Rheumatology, Franciscus Gasthuis and Vlietland Hospital, Rotterdam, the Netherlands; 5 Department of Dermatology, Franciscus Gasthuis and Vlietland Hospital, Rotterdam, the Netherlands; 6 Department of Gastroenterology and Hepatology, Franciscus Gasthuis and Vlietland Hospital, Rotterdam, the Netherlands

**Keywords:** Immune mediated inflammatory diseases, Immunocompromised patients, Vaccination care, Vaccine coverage

## Abstract

**Background:**

Patients with Immune Mediated Inflammatory Diseases (IMIDs) using immunosuppressive therapy are at increased risk of infections, including vaccine-preventable infections. In this study, we aimed to evaluate whether patients with IMIDs on systemic immunosuppressive therapy are vaccinated according to current guidelines.

**Methods:**

A survey was sent out, between August 2022 and March 2023, to all patients with IMIDs that visited the departments of dermatology, rheumatology and gastroenterology at an academic and regional hospital in Rotterdam, the Netherlands. Patient-reported vaccination status was compared to the Dutch guidelines on vaccinations in patients with chronic inflammatory diseases.

**Results:**

A total of 1,905/5,987 patients responded to the survey (response rate 32%). After exclusion of patients without systemic immunosuppressive medication, the study population comprised 1,390 patients, median age 56 years (IQR 42–66) and 41% male. Most patients (92%) had been vaccinated according to the Dutch National Immunization Program. Before starting immunosuppressive therapy, 2% of the patients who were still considered at risk according to the Dutch guideline were vaccinated for measles, and 4% for diphtheria/tetanus/polio (DT-IPV). Additionally, 62% of patients received an annual influenza vaccine, 16% received a five-yearly pneumococcal vaccine, and 91% were fully vaccinated against COVID-19.

**Conclusion:**

Patients with IMIDs on immunosuppressive therapy are not vaccinated in accordance with the guidelines. Implementation strategies to improve the vaccination rates for patients with IMIDs should specifically focus on vaccinating against measles and diphtheria/tetanus/polio, and periodic vaccination against pneumococcal and influenza infections.

**Supplementary Information:**

The online version contains supplementary material available at 10.1007/s15010-024-02373-z.

## Introduction

Chronic Immune Mediated Inflammatory Diseases (IMIDs) refer to a group of inflammatory diseases that can affect different organs, e.g. psoriasis, inflammatory bowel disease (IBD) and rheumatoid arthritis (RA). Patients with IMIDs are frequently treated with immunomodulators, small molecules or biologics. Immunosuppressive therapy is associated with an increased risk for severe infections [1]. 

Several infections may be prevented by vaccinating against these infections, including the highly prevalent infections with influenza virus and Streptococcus pneumoniae. A higher incidence of influenza infections has been reported in patients with RA compared to matched controls (409 vs. 306 cases per 100,000 patient-years), and a 2.8-fold increase in incidence of complications [2]. Similarly, the incidence risk rate of an influenza infection was 1.54 and a higher hospitalization rate (5.4% vs. 1.85%) was reported for patients with IBD compared to non-IBD [3]. For pneumococcal infections, the reported incidence of invasive pneumococcal disease (IPD) is higher in patients with chronic inflammatory diseases compared to healthy controls (65 vs. 10 per 100,000 persons) with a mortality rate ranging from 0 to 10% [4], and a 2.1–2.7 fold increased when using immunosuppressive therapy [5]. National and international guidelines on standard immunization schedules for patients with IMIDs are based on these risks. [6–9]

Significant barriers for vaccinating are seen for both patients and the treating physicians. The barriers for patients include limited awareness regarding recommended vaccinations, concerns about potential side effects and high costs due to insufficient funding and reimbursement [10–13]. Furthermore, treating physicians face challenges in providing patients with relevant information and prescription of vaccinations as a result of insufficient knowledge concerning immunization schedules, an abundance of guidelines, the insufficient reimbursement for patients and time constraints during outpatient consultations. Finally, vaccination care is hampered by the involvement of many different stakeholders, e.g. patients, healthcare providers in primary and secondary care, pharmaceutical companies and healthcare institutions, which often results in inadequate communication among healthcare providers regarding the responsibility for prescription and administration of vaccinations [11, 14]. 

The impact of above-mentioned barriers to vaccinate patients with IMIDs on vaccination rates is unknown. The aim of this study is to assess patient-reported vaccination status in patients with IMIDs on immunosuppressive treatment. The secondary aim is to explore how patients had been informed about the recommended vaccinations.

## Methods

### Study design

This observational, non-interventional study was performed at the Erasmus MC academic hospital (EMC) and the Franciscus Gasthuis & Vlietland regional teaching hospital (FGV) in Rotterdam, the Netherlands. Patients with a confirmed IMID diagnosis who were treated at the outpatient clinic between 2021 and 2023, were selected by a data team based on Diagnosis Treatment Combination codes (DTC). By means of a DTC performance code established by the Dutch Healthcare Authority (NZa), a DTC describes the closed and validated process of specialist medical care. The online questionnaire was sent out to patients of the EMC during the period of August to October 2022, and FGV during January to March 2023. Selected patients received the online questionnaire, after digital informed consent was provided. Patients received a reminder after 4 weeks by e-mail. The questionnaire was designed using a secure web-based system (Castor Electronic Data Capture). Medication information was obtained from medical records and included in the questionnaire. For analyses, only patients on systemic immunosuppressive medication were selected.

### Patient population

Patients aged 18 years or older, with a confirmed IMID diagnosis and who received immunosuppressive therapy at the outpatient clinic were included. Specific IMID diagnoses were selected at the departments of (a) dermatology, i.e. atopic eczema, hidradenitis suppurativa (HS), psoriasis, pemphigoid/pemphigus, (b) gastroenterology, i.e. Crohn’s disease, ulcerative colitis, and (c) rheumatology, i.e. psoriatic arthritis, RA, spondyloarthropathy.

### Questionnaire

Close-ended questions were asked regarding the baseline patient and disease characteristics. Vaccination status was evaluated for vaccinations within the Dutch National Immunization Program (NIP) (Supplementary Table 1), COVID-19 vaccination, travel vaccinations, vaccinations before the start of immunosuppressive therapy and additional vaccinations (Supplementary Table 2). Patients were also asked how they had been informed about current vaccination care, and if they were satisfied with the information they had received. If patients were dissatisfied, they were asked how information on vaccination care could be improved.

### Guidelines

The patient reported vaccination status was compared to the Dutch guideline on vaccinations in patients with chronic inflammatory diseases [6]. A summary of the checklist of this guideline is depicted in Supplementary Table 3.

### Data synthesis and statistical analysis

Responses to the closed-ended questions from the questionnaire were entered in a database for descriptive analyses with IBM SPSS Statistics for Windows, version 25 (IBM Corp., Armonk, N.Y., USA). Data is presented as count and percentage for categorical variables, and median and interquartile range (IQR) for non-normally distributed variables.

## Results

### Baseline characteristics

**Fig. 1:**
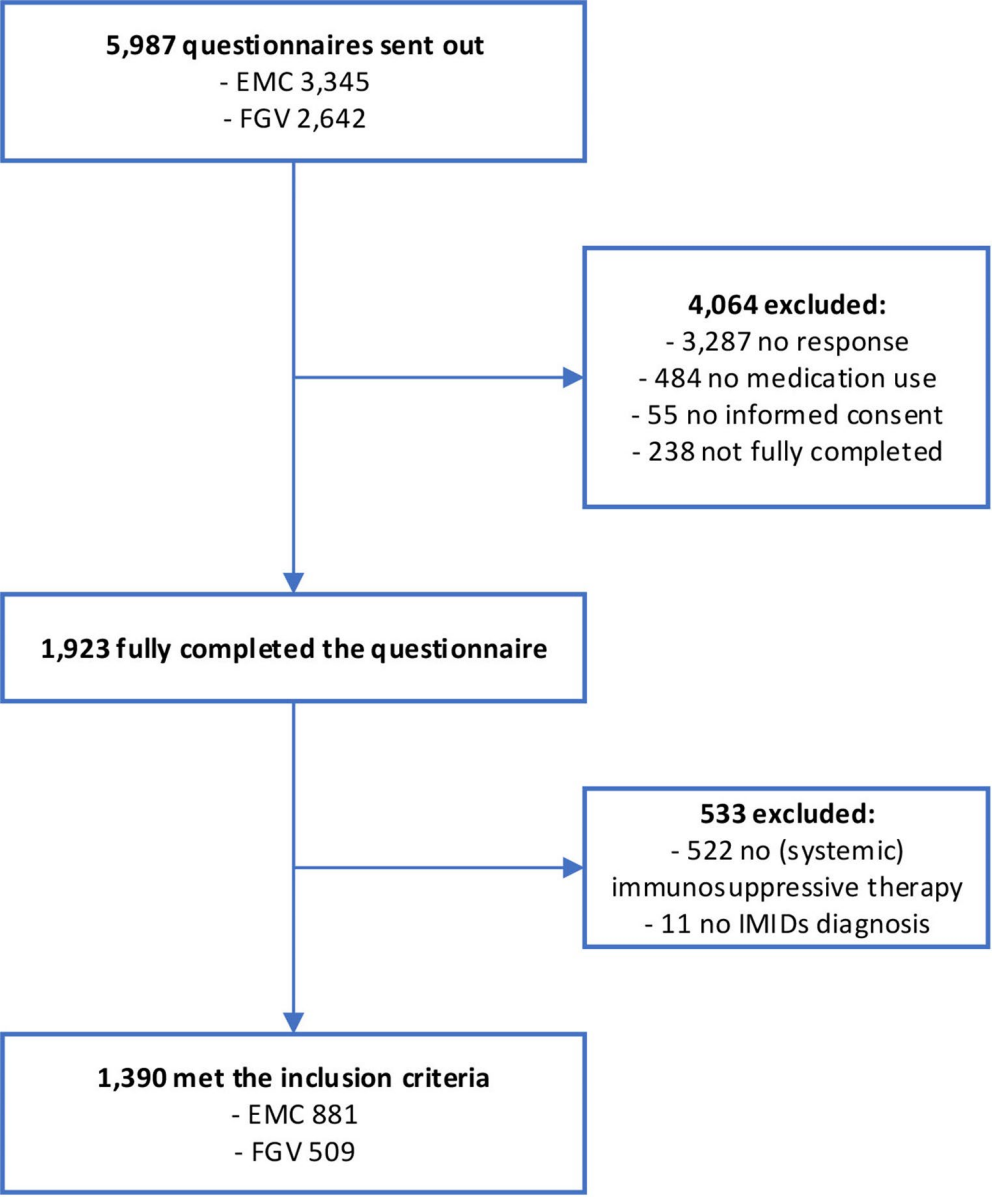
Flowchart of the selection process. Abbreviations: EMC, Erasmus MC; FGV, Franciscus Gasthuis & Vlietland; IMIDs, Immune Mediated Inflammatory Diseases

The survey was sent out to 5,987 patients with IMIDs, of whom 1,923 patients completed the questionnaire (response rate 32.1%). A total of 1,390 patients fulfilled the inclusion criteria (Fig. 1). Median age was 56 years (IQR 42–66), 40.9% was male, and 63.4% was treated at the EMC (academic hospital) vs. 36.6% at the FGV (regional hospital). Patients were treated by a dermatologist (*n* = 328, 23.6%), rheumatologist (*n* = 821, 59.1%) or gastroenterologist (*n* = 436, 31.4%), and 182 patients (13.1%) were treated for more than one diagnosis. Medication use included systemic corticosteroids (22%), immunomodulators (57.8%), JAK inhibitors (4.2%), and biologicals (62.2%) (Table 1).

**Table 1:** Baseline characteristics

		Overall (*n* = 1,390)	Dermatology (*n* = 328)	Rheumatology (*n* = 821)	Gastroenterology (*n* = 436)
**Median age**,** y (IQR)**		56 (42–66)	54 (42–64)	59 (48–69)	49 (36–61)
**Male sex**		569 (40.9)	161 (49.1)	291 (35.4)	202 (46.3)
**Median BMI**,** kg/m2 (IQR)**		25.7 (23–29.6)	27 (24.2–30.6)	26 (23.2–30.1)	24.6 (22.3–27.8)
**Born in the Netherlands**		1,259 (90.6)	291 (88.7)	741 (90.3)	409 (93.8)
**Smoking**	**Current**	176 (12.7)	65 (19.8)	97 (11.8)	39 (8.9)
	**Past**	653 (47)	156 (47.6)	411 (50.1)	193 (44.3)
	**Never**	548 (39.4)	103 (31.4)	308 (37.5)	199 (45.6)
**Education** ^**a**^	**Low**	331 (23.8)	88 (26.8)	225 (27.4)	73 (16.7)
	**Medium**	489 (35.2)	114 (34.8)	268 (32.6)	167 (38.3)
	**High**	536 (38.6)	116 (35.4)	306 (37.3)	189 (43.3)
**Work**	**Employed**	718 (51.7)	184 (56.1)	369 (44.9)	258 (59.2)
	**Unemployed**	105 (7.6)	25 (7.6)	72 (8.8)	19 (4.4)
	**Volunteer work**	63 (4.5)	17 (5.2)	40 (4.9)	22 (5)
	**Occupational disability**	212 (15.3)	52 (15.9)	128 (15.6)	80 (18.3)
	**Pensioner**	349 (25.1)	69 (21)	261 (31.8)	69 (15.8)
	**Student**	37 (2.7)	8 (2.4)	4 (0.5)	30 (6.9)
**Diagnosis**	**Atopic eczema**		22 (6.7)		
	**Hidradenitis suppurativa**		49 (14.9)		
	**Pemphigoid/pemphigus**		5 (1.5)		
	**Psoriasis**		201 (61.3)		
	**Psoriatic arthritis**			173 (21.1)	
	**Rheumatoid arthritis**			528 (64.3)	
	**Spondyloarthropathy**			84 (10.2)	
	**Crohn’s disease**				272 (62.4)
	**Ulcerative colitis**				148 (33.9)
**Diagnosis median age**,** y (IQR)**			31 (18–46)	42 (29–55)	27 (19–41)
**Medication** ^**b**^	**Systemic corticosteroids**	305 (21.9)	64 (19.5)	170 (20.7)	121 (27.8)
	*Budesonide*	*76/305 (24.9)*	*11/64 (17.2)*	*7/170 (4.1)*	*74/121 (61.2)*
	*Dexamethasone*	*28/305 (9.2)*	*9/64 (14.1)*	*14/170 (8.2)*	*11/121 (9.1)*
	*Prednisone*	*224/305 (73.4)*	*50/64 (78.1)*	*156/170 (91.8)*	*56/121 (46.3)*
	**Immunomodulators**	803 (57.8)	160 (48.8)	581 (70.8)	172 (39.4)
	*Apremilast*	*15/803 (1.9)*	*11/160 (6.9)*	*8/581 (1.4)*	*2/172 (1.2)*
	*Calcineurin inhibitors*	*28/803 (3.5)*	*10/160 (6.3)*	*5/581 (0.9)*	*16/172 (9.3)*
	*Dimethyl fumarate*	*17/803 (2.1)*	*17/160 (10.6)*	*2/581 (0.3)*	*0/172 (0)*
	*Hydroxychloroquine*	*257/803 (32)*	*19/160 (11.9)*	*246/581 (42.3)*	*9/172 (5.2)*
	*Leflunomide*	*69/803 (8.6)*	*9/160 (5.6)*	*69/581 (11.9)*	*0/172 (0)*
	*Methotrexate*	*474/803 (59)*	*87/160 (54.4)*	*410/581 (70.6)*	*44/172 (25.6)*
	*Mycophenolic acid*	*10 /803 (1.2)*	*5/160 (3.1)*	*4/581 (0.7)*	*2/172 (1.2)*
	*Purine analogues*	*127/803 (15.8)*	*15/160 (9.4)*	*17/581 (2.9)*	*112/172 (65.1)*
	**JAK inhibitors**	59 (4.2)	6 (1.8)	48 (5.8)	16 (3.7)
	**Biologicals**	864 (62.2)	219 (66.8)	466 (56.8)	313 (71.8)
	*Abatacept*	16/864 (1.9)	2/219 (0.9)	16/466 (3.4)	1/313 (0.3)
	*Anti-TNF-alpha agents*	621/864 (71.9)	146/219 (66.7)	360/466 (77.3)	217/313 (69.3)
	*Interleukin inhibitors*	211/864 (24.4)	82/219 (37.4)	95/466 (20.4)	66/313 (21.1)
	*IL-4/13*	*3/211 (1.4)*	*2/82 (2.4)*	*1/95 (1.1)*	*0 (0)*
	*IL-6*	*38/211 (18)*	*0 (0)*	*38/95 (40)*	*0 (0)*
	*IL-12/23*	*103/211 (48.8)*	*43/82 (52.4)*	*14/95 (14.7)*	*65/66 (98.5)*
	*IL-13*	*1/211 (0.5 )*	*1/82 (1.2)*	*0 (0)*	*0 (0)*
	*IL-17*	*59/211 (28)*	*30/82 (36.6)*	*43/95 (45.3)*	*2 (3)*
	*IL-23*	*17/211 (8.1)*	*13/82 (15.9)*	*5/95 (5.3)*	*0 (0)*
	*Omalizumab*	*1/864 (0.1)*	*1/219 (0.5)*	*0 (0)*	*0 (0)*
	*Rituximab*	*20/864 (2.3)*	*3/219 (1.4)*	*18/466 (3.9)*	*0 (0)*
	*Vedolizumab*	56/864 (6.5)	4/219 (1.8)	1/466 (0.2)	56/313 (6.5)

Note: ^a^ Education level is according to definitions from the Dutch Central Bureau of Statistics (CBS) [15]. Low is primary education, vmbo and mbo1; medium is havo, vwo and mbo; high is hbo, wo bachelor, wo master and doctor. ^b^ Medication is divided in subgroups: systemic corticosteroids, Immunomodulators, JAK inhibitors and biologicals. calcineurin inhibitors include cyclosporine and tacrolimus; purine analogues include azathioprine, mercaptopurin and tioguanine; JAK inhibitors includes abrocitinib, baricitinib, filgotinib, tofacitinib, upadacitinib; anti-TNF alpha agents include adalimumab, certolizumab, etanercept, golimumab and infliximab; interleukin inhibitors includes IL-4/13 (dupilumab), IL-6 (sarilumab, tocilizumab), IL-12/23 (ustekinumab), IL-13 (tralokinumab), IL-17 (bimekizumab, brodalumab, ixekizumab, secukinumab) and IL-23 (guselkumab, risankizumab)

Abbreviations: IQR, interquartile range; kg/m2, kilograms per square meter; n, number; y, year

### Vaccination status according to the Dutch guideline

**Fig. 2:**
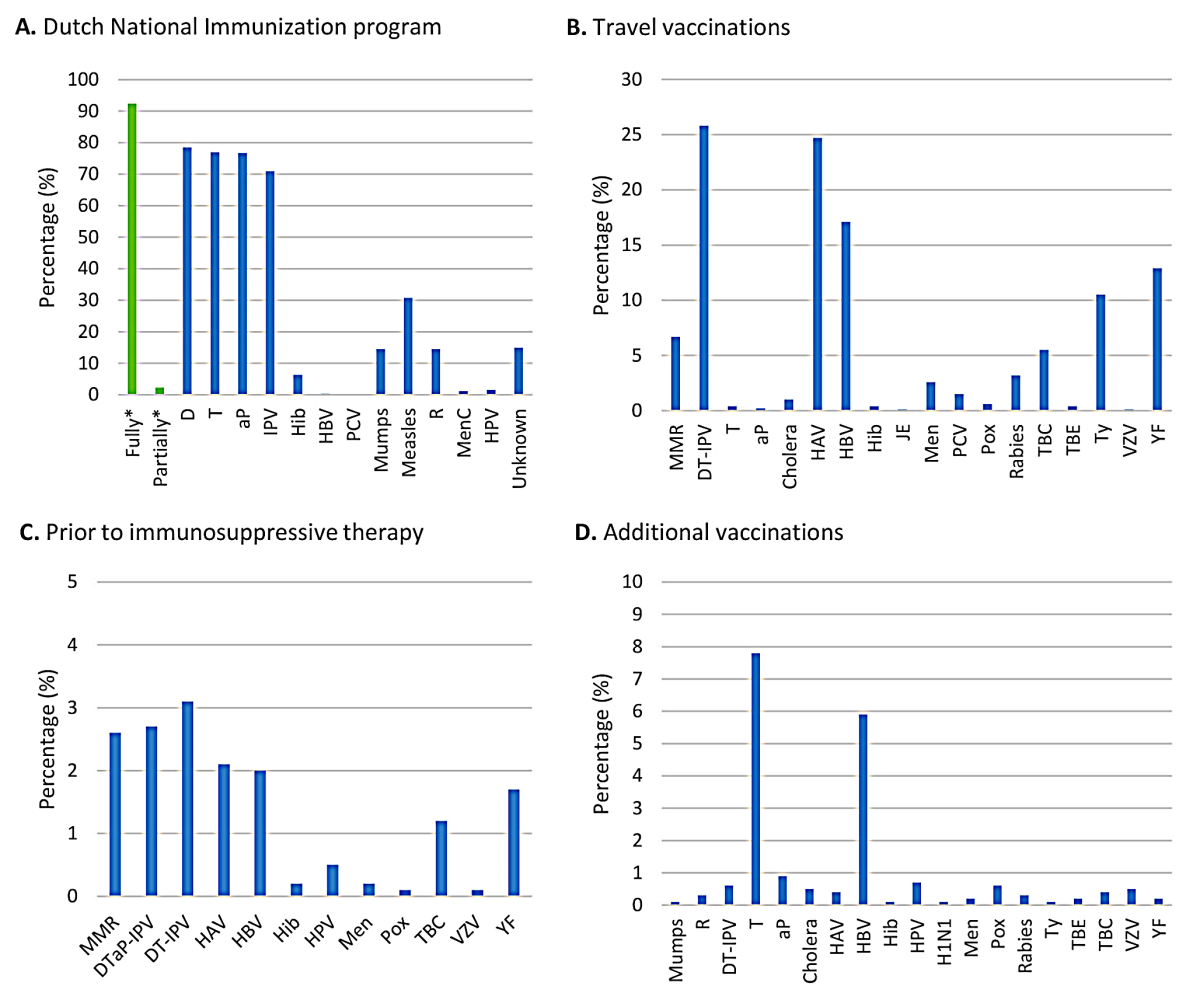
Vaccination status in patients with IMIDs (*n* = 1,390). Note: the vaccination rates are described as a percentage of the total population (*n* = 1,390). The different panels describe the vaccination status according to patients self-report for (**A**) within the Dutch National Immunization Program, (**B**) Travel vaccinations, (**C**) Vaccinations prior to initiation of immunosuppressive therapy (**D**) additional vaccinations^a^. * = fully vaccinated describes patients which received the childhood vaccinations based on birth year and implementation date. Partially vaccinated describes the patients who only received a subset of the childhood vaccinations. ^a^ = Other reasons includes e.g. work-related vaccinations, catch-up vaccinations for people who missed the routine children vaccinations according to the NIP, and after an abrasion or bite wound. Abbreviations: aP, Whooping cough; D, Diphtheria; HAV, Hepatitis A; HBV, Hepatitis B; Hib, Haemophilus influenzae type b; HPV, Human Papillomavirus; H1N1, Influenza (Mexican/Spanish flu); IPV, Polio; JE, Japanese encephalitis; Men, Meningococcal; MenC, Meningococcal type C; MMR, Mumps, Measles and Rubella; PCV, Pneumococcal; Pox, Smallpox; R, Rubella; T, Tetanus TBC, Tuberculosis; TBE, Tick-borne encephalitis; Ty, Typhoid fever; VZV, Varicella Zoster virus; YF, Yellow fever

Of all patients, 92.4% (*n* = 1,285) had received the childhood vaccinations in accordance with the NIP, and 2.3% (*n* = 32) had received a subset of these vaccinations (Fig. 2A). Of the patients who were not fully vaccinated for diphtheria/tetanus/polio (DT-IPV), 3.9% (n/*N* = 12/305) had received the diphtheria/tetanus/wheezing cough/polio (DT(aP)-IPV) vaccination before the start of immunosuppressive therapy. 2.4% (n/*N* = 8/331) of the patients at risk of measles infection received the Mumps/Measles/Rubella (MMR) vaccination prior to immunosuppressive therapy, and 7.6% (n/*N* = 25/331) of those at risk received the vaccination prior to international travel. In addition, of all patients, 2.9% (*n* = 41) received a booster DT(aP)-IPV vaccination prior to immunosuppressive therapy, and 6.9% (*n* = 96) received a booster MMR vaccination prior to immunosuppressive therapy or for travelling abroad.

61.6% of all patients received the annual influenza vaccination, and 16.3% received the pneumococcal vaccination every five years. Influenza and pneumococcal vaccination rates were higher among elderly patients who surpassed the age limit for the national vaccination program (> 60 years for influenza and > 66 years for pneumococcal). Highest vaccination rates were observed in patients treated by a rheumatologist, and when treated in a regional hospital. Analyzing vaccination rates based on diagnosis showed that both the influenza and pneumococcal vaccination rates were highest in patients with RA (68.8%, and 25%, respectively). Lowest vaccination rates were observed in patients with HS for both the influenza vaccination (32.7%) and pneumococcal vaccination (0%) (Table 2).

**Table 2:** Vaccination status for influenza and pneumococcal vaccines according to diagnosis

		Overall (*n* = 1,390)	HS (*n* = 49)	Psoriasis (*n* = 201)	CD (*n* = 272)	UC (*n* = 148)	PsA (*n* = 173)	RA (*n* = 528)	SpA (*n* = 84)
**Influenza**	**Yearly**	856 (61.6)	16 (32.7)	110 (54.7)	169 (62.1)	91 (61.5)	103 (59.5)	363 (68.8)	50 (59.5)
	**Occasionally**	113 (8.1)	3 (6.1)	13 (6.5)	25 (9.2)	16 (10.8)	12 (6.9)	39 (7.4)	8 (9.5)
	**Never**	421 (30.3)	30 (61.2)	78 (38.8)	78 (28.7)	41 (27.7)	58 (33.5)	126 (23.9)	26 (31)
	Age > 60 y*	425 (78)	2 (40)	57 (74)	48 (76.2)	35 (83.3)	53 (74.6)	238 (81.5)	12 (85.7)
	Age < 60 y*	431 (50.9)	14 (31.8)	53 (42.7)	121 (57.9)	56 (52.8)	50 (49)	125 (53)	38 (54.3)
**Pneumococcal**	**5-Yearly**	226 (16.3)	0 (0)	23 (11.4)	26 (9.6)	24 (16.2)	24 (13.9)	132 (25)	8 (9.5)
	**Occasionally**	24 (1.7)	0 (0)	1 (0.5)	4 (1.5)	1 (0.7)	6 (3.5)	8 (1.5)	0 (0)
	**Never**	1,140 (82)	49 (100)	177 (88.1)	242 (89)	123 (83.1)	143 (82.7)	388 (73.5)	76 (90.5)
	Age > 66 y*	173 (51.6)	0 (0)	16 (40)	18 (58.1)	18 (81.1)	19 (42.2)	106 (53.3)	5 (62.5)
	Age < 66 y*	53 (5)	0 (0)	7 (4.3)	8 (3.3)	6 (4.8)	5 (3.9)	26 (7.9)	3 (3.9)

Note: * = number of patients who received the yearly or five-yearly vaccination within the subgroup. Subgroups are based on the national program by the RIVM. The age limit was > 60 years old for the influenza vaccination, and > 66 years old for the pneumococcal vaccination

Abbreviations: CD, Crohn’s disease; HS, hidradenitis suppurativa; n, number; PsA, psoriatic arthritis; RA, rheumatoid arthritis; SpA, spondyloarthropathy; UC, ulcerative colitis; y, year

Finally, 92.6% (*n* = 1,287) of all patients were fully vaccinated with the COVID-19 vaccination, with 81.7% having received at least one booster vaccination (Table 3). Among all patients, 597/1,390 patients received travel vaccinations (Fig. 2B). 122/1,390 patients received vaccinations prior to the start of immunosuppressive therapy (Fig. 2C). Additional vaccinations for other reasons (e.g. work-related, after a wound or catch-up childhood vaccinations) were reported by 182/1,390 patients (Fig. 2D).

**Table 3:** COVID-19 vaccination status

		Overall (*n* = 1,390)
**Not vaccinated**		87 (6.3)
**Partially vaccinated**		16 (1.2)
**Fully vaccinated without booster**		236 (17)
**Fully vaccinated with 1 booster**		564 (40.6)
**Fully vaccinated with ≥ 2 boosters**		487 (35)
**Type of vaccination**	**Pfizer**	1,071 (82.2)
	**Moderna**	652 (50)
	**Astra Zeneca**	28 (2.1)
	**Johnson & Johnson**	40 (3.1)

Note: Fully vaccinated is defined as either after a second dose in a two-dose series (e.g. Pfizer-BioNTech, Moderna) or after a first dose in a single-dose series (e.g. Janssen/Johnson & Johnson)

Abbreviations: n, number

### Patient education

**Fig. 3:**
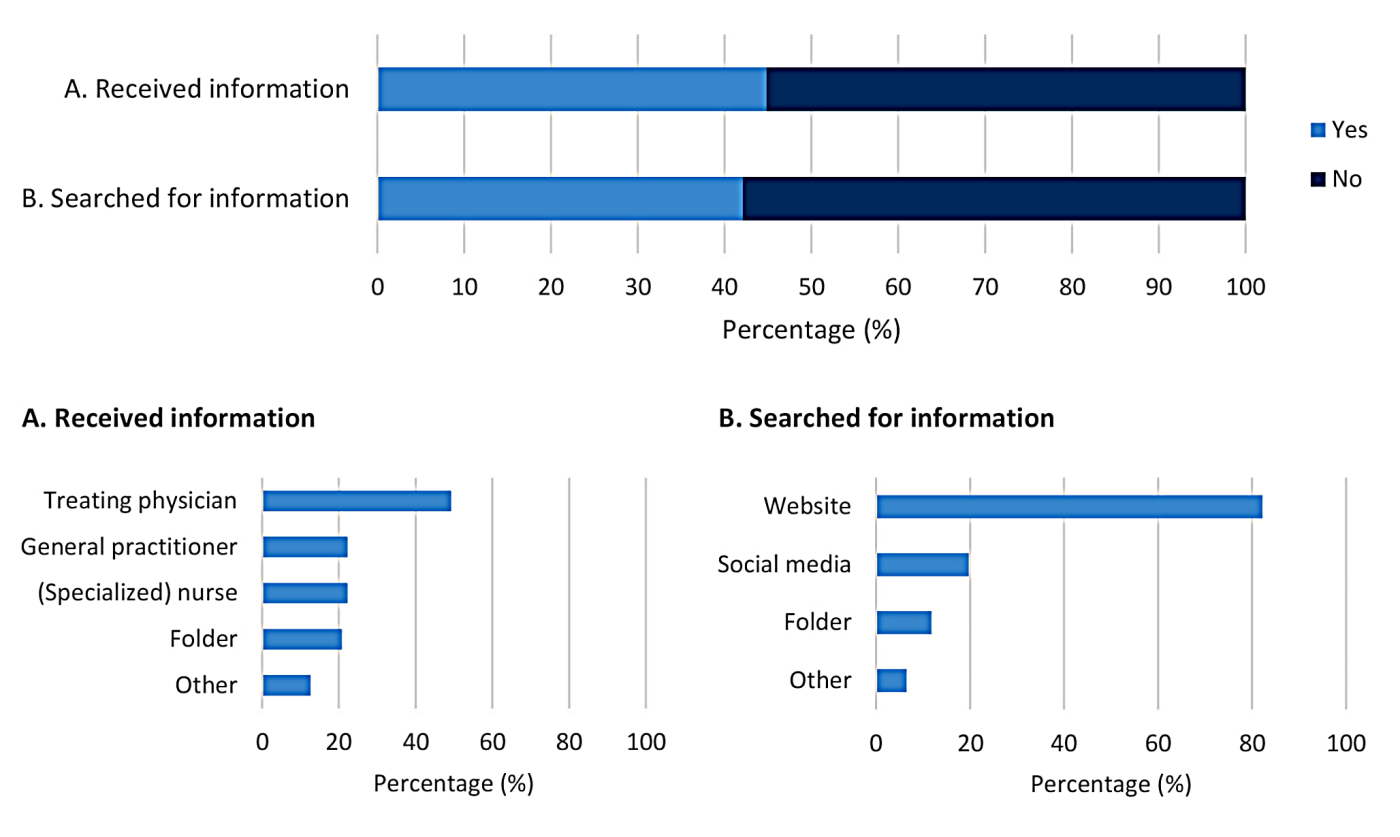
Methods of patient education. Note: the information rates are described as a percentage of the patients who received/searched for information

**Fig. 4:**
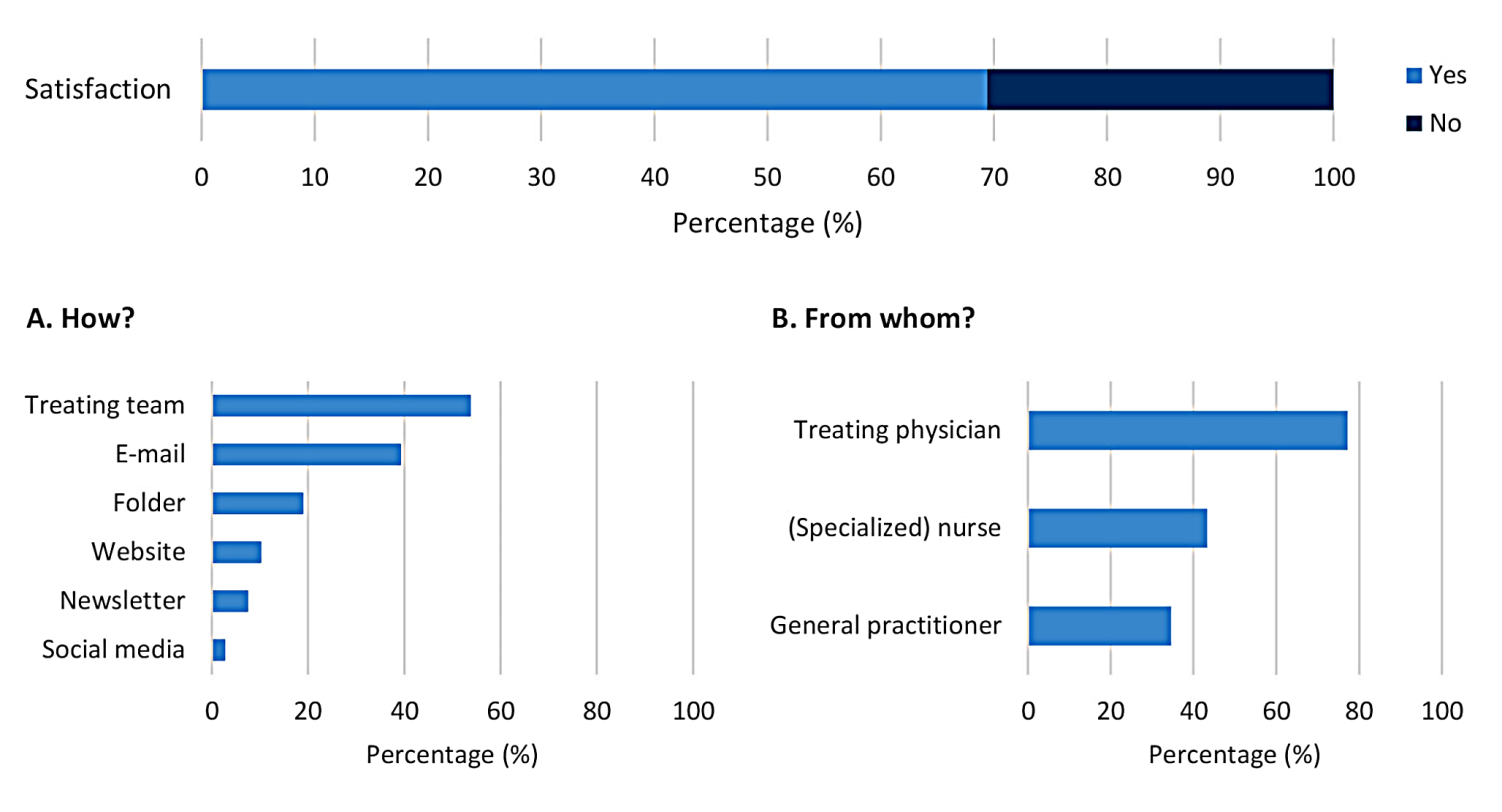
Patient preferences for receiving information. Note: the preference rates are described as a percentage of the patients who were dissatisfied with the information they received

Regarding patient education about recommended vaccinations in general, 69.4% (*n* = 965) reported being satisfied with the received information. Of these patients, 61.9% (*n* = 597) received information from their medical team, 48.7% (*n* = 470) searched for information themselves, and 25.1% (*n* = 242) neither received nor searched for information. Figure 3 illustrates the different ways patients were informed about the required vaccinations before initiating immunosuppressive therapy. Of the patients who were dissatisfied with the information they received, most patients would prefer to receive information from their medical team (53.6%; *n* = 228), and specifically their treating physician (77.2%; *n* = 328) (Fig. 4).

28.6% (*n* = 398) of patients recalled having discussed the recommended vaccinations before the start of immunosuppressive therapy. No difference in advising vaccinations prior to immunosuppressive therapy was observed between the academic and regional teaching hospital or between different treating specialties.

## Discussion

This observational study shows a high vaccination rate with regard to the vaccinations according to the NIP, COVID-19 vaccinations and travel vaccinations in patients with IMIDs on systemic immunosuppressive therapy, whereas the vaccination rates prior to initiating immunosuppressive therapy as well as influenza and pneumococcal vaccination were considerably lower. This study identifies specific components of the vaccination program that require implementation strategies to improve the vaccination rates for patients with IMIDs on immunosuppressive treatment who remain at risk for vaccine-preventable infections in the Dutch population. Most importantly, a focus is required on pneumococcal vaccination (unvaccinated population up to 84%), influenza (38%), measles (21%), and DT-IPV (21%).

The uptake of influenza vaccinations and pneumococcal vaccinations shows a high variation according to available literature. A recent systematic review and meta-analysis on interventions to increase vaccine uptake in patients with IBD and RA, shows that the range of uptake for influenza ranges from 4 to 82% and for pneumococcal from 0.3 to 80% [16]. Differences in vaccination rates between the studies are probably explained by the differences in the organization of the vaccination care, access to the vaccines, primary documentation, patient education, and patient and physicians attitude towards vaccinations. To improve vaccination uptake all these aspects require consideration.

Explanations for the high vaccination uptake (> 90%) for vaccinations included in the NIP and for the COVID-19 vaccination in our study include a nationally centralized program with one clear stakeholder, the Dutch Ministry of Health, Welfare and Sport (RIVM), and patient education. The NIP is offered to all children in the Netherlands and new parents automatically receive information about the vaccinations and an invitation for their children [17]. Moreover, during the COVID-19 pandemic, a national vaccination program was swiftly implemented and patients received an invite for the vaccination based on age and comorbidities. Extensive media coverage emphasizing the importance of COVID-19 vaccinations possibly contributed to the high vaccination uptake [18]. Learning from these programs, including centralized organization, funding and education may benefit specific programs in patients with IMIDs.

These important aspects probably also explain the higher vaccination rates among older IMID patients as compared to younger patients, since all individuals above a specific age limit (> 60 years for influenza and > 66 years for pneumococcal) are invited to the national program to receive an influenza or pneumococcal vaccination. This might partially explain differences observed in this study between the overall vaccination rates in patients with HS versus patients with RA. Implementation strategies for a vaccination program for younger patients are required since our study showed that only half of these patients received an annual influenza vaccination and only 5% received the five-yearly pneumococcal vaccination. This implementation program may also focus on the MMR and DT-IPV vaccination. Further evaluation of the indications for the newer recombinant herpes zoster vaccination for patients with IMIDs are required before implementation. Current data and guidelines are equivocal on this indication and recommend to consider this vaccination on individual basis [6]. 

The findings highlight key factors influencing the implementation of vaccination guidelines. Adequate patient education, well-defined stakeholder collaborations, and comprehensive national vaccination programs contribute to high vaccination rates. However, when the responsibility lies within the medical team, vaccination rates are considerably lower. Current education is insufficient and recommended vaccinations prior to immunosuppressive therapy are often not discussed by health care providers. In this study we observed considerable differences between vaccination rates in specific departments, such as a higher vaccination rate in patients with RA. These variations probably result from differences in the logistical implementation and execution of vaccination care across departments and hospitals. For instance, the higher vaccination rate observed among patients with RA could potentially be due to comprehensive patients education facilitated by specialized nurses. Overall, patients with IMIDs have some general knowledge about vaccinations (e.g. efficacy, costs, protection for severe diseases), but understanding specific details like vaccine efficacy and contraindications with immunosuppressive therapy is limited, varying with education levels [19]. This underlines the need for thorough patient education to improve vaccination care for patients with IMIDs. Studies show that effective education improves vaccination uptake by raising awareness among both patients and health care providers [20–23]. Sustained education efforts are crucial for long-term vaccination uptake, addressing vaccine hesitancy, and considering early education after diagnosis, preferably before immunosuppressive treatment is contemplated. Our study underscores the vital role of the medical team, particularly treating physicians, in providing this information to patients regarding vaccinations.

Patients and physicians attitude towards vaccinating are not taken into account in this study, which might contribute to a lower vaccination uptake. Surprisingly, a quarter of the patients who were satisfied with the information about the recommended vaccinations did not receive any information at all, which might indicate a group of patients with a contentedly ignorant attitude. From the physicians point of view, the recommendations may vary among specialists because of differing assessments of the necessity for these vaccinations, possibly due to the perceived relatively low prevalence of severe infections. Further research is needed to assess the acceptance of the guidelines by treating physicians.

A few limitations of this study need to be taken into account. First, it is important to note that this study is based on self-reported vaccination status by patients. This may have led to a potential recall bias. Unfortunately, the absence of a national registration system for vaccinations leaves limited options for evaluating vaccination status and poses a challenge for healthcare providers in accurately determining the vaccination status of their patients. Secondly, the travel and additional vaccinations may introduce potential bias, as it involves only patients who were conscious of the necessity for vaccinations. The exclusion of patients who were unaware of recommended vaccinations may result in an overestimation of the overall vaccination rate. Finally, it is important to note that this study was conducted in the Netherlands, which limits the generalizability of the findings to in other countries. The variation in vaccination programs and care across different countries need to be taken into account.

## Conclusion

Patients with Immune Mediated Inflammatory Diseases on immunosuppressive therapy are not vaccinated in accordance with the current guidelines. Patients are thus insufficiently protected against vaccine-preventable infections, including influenza and pneumococcal infections. Important facilitators include patient education, well-defined stakeholder collaborations and comprehensive national vaccination programs. Barriers persist within the medical team, highlighting the need for better implementation strategies of current vaccination guidelines and effective education for patients and physicians.

## Electronic supplementary material

Below is the link to the electronic supplementary material.

Supplementary Material 1

## Data Availability

Data are available on reasonable request.
