# Supplementary material for: Patients with immune mediated inflammatory diseases are insufficiently protected against vaccine-preventable infections
Source: Infection. 2024 Aug 22;53(1):317–27. doi: 10.1007/s15010-024-02373-z (PMC11825543; doi:10.1007/s15010-024-02373-z)
Supplement: Supplementary file 1 — Supplementary Material 1 [file 15010_2024_2373_MOESM1_ESM.docx]

**Patients with Immune Mediated Inflammatory Diseases are insufficiently protected against vaccine-preventable infections**

N. van de Pol^1^, C.J. van der Woude^1^, M. Vis,^2^ M.B.A. van Doorn,^3^ S.L. Schrauwen,^4^ F. Cetinözman-Teunissen,^5^ R.L. West^6^ A.C. de Vries^1^

1. Department of Gastroenterology and Hepatology, Erasmus University Medical Center, Rotterdam, the Netherlands

2. Department of Rheumatology, Erasmus University Medical Center, Rotterdam, the Netherlands

3. Department of Dermatology, Erasmus University Medical Center, Rotterdam, the Netherlands

4. Department of Rheumatology, Franciscus Gasthuis and Vlietland Hospital, Rotterdam, the Netherlands

5. Department of Dermatology, Franciscus Gasthuis and Vlietland Hospital, Rotterdam, the Netherlands

6. Department of Gastroenterology and Hepatology, Franciscus Gasthuis and Vlietland Hospital, Rotterdam, the Netherlands

**Correspondence:**

Annemarie C. de Vries, MD PhD

Department of Gastroenterology and Hepatology, Erasmus University Medical Center

Dr. Molewaterplein 40, 3015 CE Rotterdam, Room Na-618, The Netherlands

Email address: [a.c.devries@erasmusmc.nl](mailto:a.c.devries@erasmusmc.nl)

**SUPPLEMENTARY TABLES AND FIGURES**

| **Age** | **Injection 1** | **Injection 2** | **Implementation dates** |
| --- | --- | --- | --- |
| **3 months** | DTaP-IPV-Hib-HBV | PCV | D, 1957; T, 1954; aP, 1957; IPV, 1957; Hib, 1993; HBV, 2011; PCV, 2006  Combination vaccine DTaP-IPV since 1962; Hib since 2003 added to combination vaccine; HBV since 2011 added to combination vaccine |
| **5 months** |  |  |  |
| **11 months** |  |  |  |
| **14 months** | MMR | MenACWY | B, 1987; M, 1976; R, 1987; MenC, 2002; MenACWY, 2018  Combination vaccine MMR since 1987 |
| **4 years** | DTaP-IPV |  |  |
| **9 years** | DT-IPV | MMR |  |
| **10 years** | HPV | HPV | HPV for girls since 2010; HPV for boys since 2022 |
| **14 years** | MenACWY |  |  |

**Supplementary table 1.** Vaccination schedule according to the Dutch National Immunization Program with implementation dates.(1)

Note: The Dutch National Immunization Program (Rijksvaccinatieprogramma, NIP) is offered to all children in the Netherlands. This program contains a standard vaccination schedule against 12 infectious diseases.

Abbreviations: DT(aP)-IPV, Diphtheria, Tetanus, Whooping cough and Polio; HBV, Hepatitis B; Hib, Haemophilus influenza type B; HPV, Human Papillomavirus; Men, Meningococcal disease; MMR, Mumps, Measles and Rubella; PCV, Pneumococcal disease.

**Supplementary table 2.** Overview of the vaccinations in the questionnaire.

| **COVID-19 vaccination** | **Travel vaccinations** | **Vaccinations before immunosuppressive therapy** | **Other vaccinations** |
| --- | --- | --- | --- |
| Pfizer | Typhoid fever | MMR | Yearly influenza |
| Moderna | MMR | DT(aP)-IPV | Five-yearly pneumococcal |
| Astra Zeneca | DTP | YF | HBV |
| Johnson&Johnson | Yellow fever | HAV | Tetanus |
|  | JE | HBV | Varicella Zoster |
|  | HAV | HPV |  |
|  | HBV | TBC |  |
|  | Men |  |  |
|  | Rabies |  |  |
|  | TBC |  |  |
|  | TBEV |  |  |

Note: The vaccination against COVID-19 has been available in the Netherlands since January 2021. Currently the standard vaccination series and multiple booster vaccinations are offered to all residents in the Netherlands.

Abbreviations: DT(aP)-IPV, Diphtheria, Tetanus, Whooping cough and Polio; HAV, Hepatitis A; HBV, Hepatitis B; HPV, Human Papillomavirus; JE, Japanese encephalitis; Men, Meningitis; MMR, Mumps, Measles and Rubella; TBC, Tuberculosis; TBEV, Tick-Borne Encephalitis; TF, Typhoid fever; YF, Yellow fever.

**Supplementary table 3.** Checklist for prescribers of immunosuppressive therapy from the Dutch guideline on vaccinations in patients with chronic inflammatory diseases.(2)

| **Vaccinations** | **Who and what** | **Comments** |
| --- | --- | --- |
| **Need for travel vaccinations** | Any travel plans now or in the future? | Refer to (travelers’) vaccination center for indication especially in relation to yellow fever vaccine (contraindicated during immunosuppressive therapy). |
| **Check NIP vaccinations** | Every patient before starting immunosuppressive therapy.  Request vaccination status according to the NIP. In case of unknown vaccination history e.g. grew up abroad or may not have had childhood vaccinations: check serology of measles, tetanus, yellow fever, hepatitis A and B, varicella, in addition to the other protocol determinations for previous exposure to infections. | Complete missing vaccinations according to NIP catch-up schedule.   - Measles: one-time MMR - DT(aP)-IPV:   - If not/partially, three   vaccinations DT(aP)-IPV   - - Ten-yearly a booster vaccine - Yellow fever: if titer present no revaccination required (only in specific medical risk groups) |
| **Measles** | For persons born in the Netherlands:  Check serology only if unclear about passed measles infection, birth cohort from 1965-1975 or if only one-time measles vaccination (born 1976-1977).  Positive: no vaccination required.  Negative: one-time MMR. | At least 4 weeks before starting immunosuppressive therapy. Consult with clinical expert regarding contraindication, possible exception with selected immunosuppressive medication or low doses.   - Assumption is that everyone born before 1965 has experienced measles: no need for titer determination or vaccination with MMR. - In persons born 1965-1976 always serology because they have had no measles vaccination and little natural immunity due to little circulation from 1965. - Persons born 1976 and 1977: measles vaccination only once in NIP. Perform serology because possibly negative after 1 vaccination. |
| **Influenza** | Every patient with immunosuppressive therapy and untreated SLE-patients | - Universal yearly recommendation for influenza vaccination - Exception during (or 6-12 months after anti-CD20 (Rituximab) |
| **Pneumococcal** | Every patient with immunosuppressive therapy | - Universal recommendation for PCV13 vaccination followed by PPV23 five yearly - Exception during (or 6-12 months after anti-CD20 (Rituximab) |
| **Varicella Herpes Zoster** | No recommendation for vaccination | Individual consideration: determine serology if patients have not had chickenpox or if it is unknown whether they have experienced chickenpox. IgG-positive: vaccination with zoster vaccine. Contraindication Zostavax during immunosuppressive therapy.   - If necessary, discuss possibility of vaccination with Shingrix - Universal recommendation lacking |

Abbreviations: DT(aP)-IPV, Diphtheria, Tetanus, Whooping cough and Polio; MMR, Mumps, Measles and Rubella; NIP, National immunization program.

1. Dutch National Immunisation Programme: Dutch Ministry of Health, Welfare and Sport; [Available from: <https://rijksvaccinatieprogramma.nl/english>.

2. Vaccinatie bij chronisch inflammatoire aandoeningen: Dutch Ministry of Health, Welfare and Sport; [Available from: <https://lci.rivm.nl/richtlijnen/vaccinatie-bij-chronisch-inflammatoire-aandoeningen#2-checklists-bij-vaccinatiezorg>.

**REFERENCES**
